# Does splitting a tablet obtain the accurate dose? A systematic review protocol

**DOI:** 10.1097/MD.0000000000017189

**Published:** 2019-10-18

**Authors:** Kanika Chaudhri, Madeleine Kearney, Gian Luca Di Tanna, Richard O. Day, Anthony Rodgers, Emily R. Atkins

**Affiliations:** a The George Institute for Global Health, Sydney; b Faculty of Medicine, UNSW Sydney; c Department of Clinical Pharmacology and Toxicology, St Vincent's Hospital, Darlinghurst; d Westmead Clinical School, University of Sydney, Sydney, Australia.

**Keywords:** dose accuracy, pill splitting, systematic review, tablet splitting

## Abstract

**Background:**

Physical manipulation of the manufactured dose form is a common practice, with almost a quarter of all drugs administered in primary care having their dose altered. Splitting a tablet can be advantageous as it facilitates swallowing, allows for dose flexibility and provides cost reductions. However, there are concerns these physical changes can lead to inaccurate portions resulting in significant variations from the prescribed dose. Thus, the review described in this protocol aims to summarise the literature assessing the effect of tablet splitting on dose accuracy.

**Methods:**

Relevant studies will be identified through electronic searches in databases including EMBASE, MEDLINE, CINAHL, and the Cochrane Library, from the beginning of databases until January 2020. Studies investigating any drug, where the tablet was split, will be potentially eligible. Two reviewers will independently screen studies and extract data using standardised forms. Data extracted will include general study information, characteristics of the study, intervention characteristics and outcomes. Primary outcome is to assess dose accuracy of a split tablet measured by drug content or weight variability. Assessment of risk of bias will be dependent upon study design. If deemed feasible, meta-analysis will be performed.

**Results:**

The study described within this protocol will provide a synthesis of current evidence assessing the effect of tablet splitting on dose accuracy.

**Conclusion:**

The conclusion of our study will provide evidence to judge whether splitting a tablet results in an accurate half dose.

**Ethics and dissemination:**

Ethics approval was not required for this study. The results of the systematic review described will be published in a peer-reviewed journal.

**Registration details:**

PROSPERO CRD42018106252

## 1 Introduction

Tablet splitting is a widely practiced phenomenon resulting from the need to alter and optimise medicine doses in individual patients. Almost a quarter of all drugs administered in primary care are split.^[1]^ This may be required for patients to overcome dysphagia caused by large tablets.^[2]^ It facilitates swallowing of the tablet and increases compliance as it eases consumption of the tablet.^[3,4]^ This practice can be relatively unproblematic if the patient ingests all fragments to deliver the desired dose.^[5]^ Additionally, tablet splitting is used as a cost-saving practice.^[1,6]^ For example, treatment prices for drugs administered in primary care were reduced by up to 45% especially where the price per tablet does not proportionally increase with increasing dose strength.^[1]^ Splitting for these reasons is a common part of current drug therapy.

Dose inaccuracy may be a consequence of inaccurate splitting or loss of tablet weight during the process of splitting.^[7]^ The resulting variation in drug mass and content may lead to adverse effects ranging from toxicity to loss of efficacy.^[8]^ This is especially important for drugs with dose-dependent effects or narrow therapeutic index and short half-life.^[9]^

Split fragments should comply with the content or mass uniformity requirements.^[10,11]^ Brand-specific product information is available on drug package leaflets which may include information on suitability of the specific tablet to be split. Unfortunately, this guidance is frequently disregarded.^[12]^ Additionally, hospitals often have their own specific drug formularies and accompanying drug information. However, these sources are often in conflict. This acts as a source of confusion for the patients, prescribers, pharmacists and nurses and warrants attention. There is a need for standardised documentation and information regarding tablet splitting.

There is limited guidance on tablet splitting. A Swiss study reported that official sources of drug information for the majority of scored tablets contained no explicit information on tablet splitting.^[5]^ Thus, given the variation between methods for splitting tablets such as using hands, tablet splitters and knives or scissors, and the variable physical characteristics of tablets, such as presence of a score line, can potentially produce differences in the resultant segments, and consequently, the dosage.^[13,14]^

Although dose manipulation is common practice, currently there is limited literature summarising the evidence available on splitting a tablet and obtaining the correct dose. Past reviews have focused on either a specific population, drug, or disease.^[15,16]^ The aim of this study is to summarise the literature measuring the effect on dose accuracy associated with splitting a tablet without limiting data sources to population, disease or drug specific studies.

## 2 Methods

The systematic review described within this protocol will be reported as per Preferred Reporting Items for Systematic Review and Meta-Analysis for protocol (PRISMA-P) recommendations. This study is registered on PROSPERO, an international register of systematic reviews (CRD42018106252).

### 2.1 Eligibility criteria

#### 2.1.1 Study design

We will exclude case studies, reports and letters. However, there will be no further restriction on study design to assess the effects of splitting a tablet. Data will also be used from laboratory-based tablet splitting studies where the drug was not administered to a patient as these studies can consider the weight or drug content of the split drug. Therefore, studies investigating any drug, where the tablet was split, will be potentially eligible. This review will include studies from the beginning of databases till January 2020.

Publications must contain sufficient detail to be included within the review, therefore, conference abstracts will be excluded from analysis. However, study authors will search databases for publications relating to the abstract.

#### 2.1.2 Types of participants

There will be no restriction in participant characteristics. Participants will be included regardless of their age or experience with tablet splitting.

#### 2.1.3 Types of interventions and comparators

Interventions will include manipulation of oral tablets (excluding capsules). Manipulation can include splitting, cutting or breaking tablets into smaller sections. Comparator is the whole, unbroken tablet.

#### 2.1.4 Study outcomes

The primary outcome is to assess the dose accuracy of the split drug either by weight or drug content. The secondary outcomes are to assess variation in dose accuracy between methods for splitting as well as physical characteristics, differences in health outcomes when ingesting split tablet and patient satisfaction from using the split tablet. The study must assess primary outcome to be eligible for the review.

### 2.2 Search strategy

This systematic review will involve a search of MEDLINE, EMBASE, CINAHL, and Cochrane databases. Any study published from the start of the databases prior to January 2020 will be included in the review.

The search strategy will capture studies that include key words outlined below.

1. Intervention: (tablet∗ split∗ or tablet∗ break∗ or tablet∗ cut∗ or tablet∗ manipulat∗).mp. [mp=title, abstract, original title, name of substance word, subject heading word, floating sub-heading word, keyword heading word, protocol supplementary concept word, rare disease supplementary concept word, unique identifier, synonyms]
2. Primary outcome: (pill∗ split∗ or pill∗ break∗ or pill∗ cut∗ or pill∗ manipulat∗).mp. [mp=title, abstract, original title, name of substance word, subject heading word, floating sub-heading word, keyword heading word, protocol supplementary concept word, rare disease supplementary concept word, unique identifier, synonyms]

Additionally, further studies will be obtained from scanning reference lists of included studies and citation searching of key papers. This will ensure the maximum number of relevant articles are included within the review.

### 2.3 Data management

After searching, the shortlisted articles were exported to Endnote X9 (Thomson Reuters, NY) for storage of study records, abstracts and full text articles. Articles will be stored on a password protected server-based platform that is accessible to both reviewers. At each stage of the article selection process (e.g., after consolidation of all articles prior to assessing eligibility based on title and abstract), back up files of the Endnote database will be made in order to retrace any steps as needed in the review process.

### 2.4 Selection process

Two researchers will undertake the selection of studies process separately to reduce the risk of bias. In the initial screening stage, these authors will conduct a title search and identify abstracts which potentially meet the criteria for study selection. For papers where it is unclear whether the study should be included, a further assessment against the criteria will be undertaken, using the full text of these articles. This will be done independently to reduce risk of bias. Discrepant opinions between two reviewers will be resolved in discussion with the senior author. The flow of studies through selection process, together with reasons for exclusion at the full-text review stage will be reported using a modified PRIMSA diagram.

### 2.5 Data collection process

Once the studies for inclusion are identified, information outlined in the standardised data extraction form will be collected. Data from all included studies will be extracted. The form will be piloted and optimised by the two reviewers using a subset of five randomly selected studies that satisfy the eligibility criteria. One author will independently extract data from the remainder of the included studies. The data extracted will be verified by a second reviewer.

### 2.6 Data items

The following data will be extracted from the included studies:

1. General study information: study title, study authors, year of publication, and citation.
2. Characteristics of the study: aim or objectives of the study, country in which study took place, study design, condition, and pharmacopeia referenced.
3. Participant characteristics: numbers of participants, type of participants, prior experience, and instructions given.
4. Tablet characteristics: tablet type, shape, score-line, diameter, coating, and weight.
5. Intervention characteristics: type of tablet splitting method used and any parallel interventions implemented.
6. Outcomes: result of primary outcome and statistical significance, documentation of specific quantitative and qualitative secondary outcomes of interest, risk of bias assessment, and overall study conclusion.

### 2.7 Risk of bias in individual studies

For randomised controlled trials (RCTs) included within this review, the risk of bias will be ascertained by two reviewers in parallel using The Cochrane Risk of Bias Tool.^[17]^ The Grades of Recommendation, Assessment, Development, and Evaluation (GRADE) system will be used to summarise the quality of evidence for each outcome.^[18]^

Developing a unique quality assessment tool (Table 1) using known quality tools and study specific additions will allow us to assess the quality of these studies. The items within this form will be categorised as ‘Yes’, ‘No’, or ‘Unclear’. This form draws on aspects of the Strengthening the Report of Observational Studies in Epidemiology (STROBE) checklist.^[19]^ This will ensure mapping of evidence and identification of research gaps within this field. Additionally, the items addressing data collection and analysis will be based on European, British, and United States Pharmacopoeia. The form will be piloted and adjusted prior to being applied to all studies.

**Table 1:**
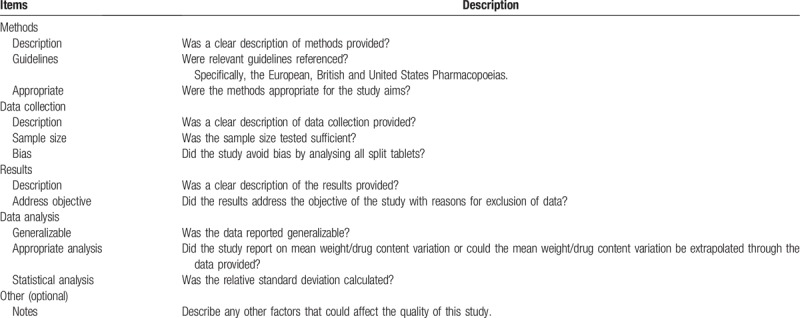
Quality appraisal tool to be used for the systematic review described within this protocol.

### 2.8 Data synthesis and analysis

Studies will be included in the data synthesis if they fulfil the eligibility criteria. Data will be presented in a descriptive narrative and supplemented with tables and figures where appropriate.

If deemed feasible (i.e., variables assessed in the selected papers are comparable and there is sufficient data) we aim to perform meta-analyses of proportions with both fixed effect (Inverse Variance) and random effects models (using the method of DerSimonian and Laird). A continuity correction of 0.5 will be applied in case of numerators equal to zero and the Wilson Score method will be used to calculate confidence intervals bounded to 0 and 1. We also intend to perform subgroup meta-analyses according to method of splitting and physical tablet characteristics. All statistical analyses will be performed using Stata 15 (StataCorp LLC, College Station, TX) with the metaprop function.^[20]^

### 2.9 Patients and public involvement

Patients and public were not involvement in writing this protocol.

## 3 Discussion

Tablet splitting is a common practice arising from the need to alter and optimise medicine doses in individual patients, but earlier studies recommend avoiding tablet splitting due to inaccuracy.^[21]^ Currently, there is limited literature summarising the evidence available on splitting a tablet and obtaining the correct dose. Consequently, the planned systematic review will synthesise evidence surrounding tablet splitting and dose accuracy.

There will be potential limitations of this review. Developing a search strategy was difficult as ‘tablet splitting’ does not have a standard term or clear definition within databases therefore, there was potential for relevant articles to be missed. The search strategy developed needed to be balanced between being sensitive, yet precise, within the papers extracted. Despite these efforts, it is possible that a large number of irrelevant references may still be retrieved which is unavoidable with search terms that are not drug or intervention specific.

Additionally, as this review is not limited to RCTs the nature of studies that may be eligible raises challenges when assessing quality of these studies. Laboratory based studies do not generally feature in systematic reviews, thus are not considered in the available quality assessment tools. We developed our own quality appraisal tool to determine the quality of such studies. Although there are limitations to this tool, systematic reviews that include a broad range of study designs either do not report on quality^[15]^ or have also undertaken a similar approach.^[16,22]^ Very few of these reviews have published their grading tool, with those that are published focusing on quality of the publication rather than the quality of the study itself.^[23]^

### 3.1 Ethics and dissemination

This study does not require ethics approval. The results of the systematic review described within this protocol will be presented at relevant conferences and published in a peer-reviewed journal. It should be noted that prescribers should follow manufacturer's instructions when splitting tablets and this review should be used as a guide. The review described within this protocol will be of interest to healthcare professionals, physicians, and pharmacists in particular, as well as people who use tablets. The methods can be used to inform future reviews exploring the effect of tablet cutting on dose accuracy. Approaches to overcome the identified challenges serve to illustrate that thorough review of current literature is required to make an assessment on the potential of splitting tablets to gain the required dose.

## Author contributions

**Conceptualization:** Emily R. Atkins.

**Data curation:** Madeleine Kearney.

**Formal analysis:** Gian Luca Di Tanna.

**Project administration:** Kanika Chaudhri.

**Supervision:** Richard O. Day, Anthony Rodgers, Emily R. Atkins.

**Writing – original draft:** Kanika Chaudhri.

**Writing – review & editing:** Gian Luca Di Tanna, Richard O. Day, Anthony Rodgers, Emily R. Atkins.

Kanika Chaudhri orcid ID: 0000-0002-3453-0769.

Gian Luca Di Tanna orcid ID: 0000-0002-5470-3567.

## References

[1] Quinzler R Gasse C Schneider A The frequency of inappropriate tablet splitting in primary care. Eur J Clin Pharmacol 2006;62:1065 73. 17024485 10.1007/s00228-006-0202-3

[2] Marquis J Schneider M-P Payot V Swallowing difficulties with oral drugs among polypharmacy patients attending community pharmacies. Int J Clin Pharm 2013;35:1130 6. 23963541 10.1007/s11096-013-9836-2

[3] Elliott I Mayxay M Yeuichaixong S The practice and clinical implications of tablet splitting in international health. Trop Med Int Health 2014;19:754 60. 24702766 10.1111/tmi.12309 PMC4285309

[4] Abu-Geras D Hadziomerovic D Leau A Accuracy of tablet splitting and liquid measurements: an examination of who, what and how. J Pharm Pharmacol 2017;69:603 12. 28028813 10.1111/jphp.12671

[5] Arnet I Hersberger KE Misleading score-lines on tablets: facilitated intake or fractional dosing? Swiss Med Wkly 2010;140:105 10. 20069474 10.4414/smw.2010.12953

[6] Gee M Hasson NK Hahn T Effects of a tablet-splitting program in patients taking HMG-CoA reductase inhibitors: analysis of clinical effects, patient satisfaction, compliance, and cost avoidance. J Manag Care Pharm 2002;8:453 8. 14613379 10.18553/jmcp.2002.8.6.453

[7] Gerber A Kohaupt I Lauterbach KW Quantification and classification of errors associated with hand-repackaging of medications in long-term care facilities in Germany. Am J Geriatr Pharmacother 2008;6:212 9. 19028377 10.1016/j.amjopharm.2008.10.005

[8] Shah RB Collier JS Sayeed VA Tablet splitting of a narrow therapeutic index drug: a case with levothyroxine sodium. AAPS PharmSciTech 2010;11:1359 67. 20740332 10.1208/s12249-010-9515-8 PMC2974142

[9] Noviasky J Lo V Luft DD Which medications can be split without compromising efficacy and safety? J Fam Pract 2006;55:707 9. 16882445

[10] Commission BP Council GM Commission GBM British pharmacopoeia. Vol 1: Her Majesty's Stationery Office; 2001.

[11] Commission EP, Medicines EDftQo, Healthcare. European pharmacopoeia. Vol 1: Council of Europe; 2010.

[12] Bosworth HB Brown JN Danus S Evaluation of a packaging approach to improve cholesterol medication adherence. Am J Manag Care 2017;23:e280 6. 29087166

[13] Verrue C Mehuys E Boussery K Is splitting tablets dangerous? Nurs Times 2011;107:23 123. 21667663

[14] Rodenhuis N De Smet PA Barends DM The rationale of scored tablets as dosage form. Eur J Pharm Sci 2004;21:305 8. 14757502 10.1016/j.ejps.2003.10.018

[15] Eserian J Lombardo M Chagas J Actual versus expected doses of half tablets containing prescribed psychoactive substances: a systematic review. Prim Care Companion CNS Disord 2018;20:10.4088/PCC.17r02211 29469240

[16] Richey RH Hughes C Craig JV A systematic review of the use of dosage form manipulation to obtain required doses to inform use of manipulation in paediatric practice. Int J Pharm 2017;518:155 66. 28040560 10.1016/j.ijpharm.2016.12.032

[17] Higgins JPT Douglas GA Gotzsche PC The Cochrane Collaboration's tool for assessing risk of bias in randomised trials. BMJ 2011;242. 10.1136/bmj.d5928 PMC3196245 22008217

[18] Guyatt G Oxman AD Akl EA GRADE guidelines: 1. Introduction—GRADE evidence profiles and summary of findings tables. J Clin Epidemiol 2011;64:383 94. 21195583 10.1016/j.jclinepi.2010.04.026

[19] Von Elm E Altman DG Egger M The Strengthening the Reporting of Observational Studies in Epidemiology (STROBE) statement: guidelines for reporting observational studies. PLoS Med 2007;4:e296. 17941714 10.1371/journal.pmed.0040296 PMC2020495

[20] Nyaga VN Arbyn M Aerts MJAoPH Metaprop: a Stata command to perform meta-analysis of binomial data. Arch Public Health 2014;72:39. 25810908 10.1186/2049-3258-72-39 PMC4373114

[21] Habib WA Alanizi AS Abdelhamid MM Accuracy of tablet splitting: comparison study between hand splitting and tablet cutter. Saudi Pharm J 2014;22:454 9. 25473334 10.1016/j.jsps.2013.12.014 PMC4246398

[22] Gülmezoglu AM Say L Betrán AP WHO systematic review of maternal mortality and morbidity: methodological issues and challenges. BMC Med Res Methodol 2004;4:16. 15236664 10.1186/1471-2288-4-16 PMC481067

[23] Hawker S Payne S Kerr C Appraising the evidence: reviewing disparate data systematically. Qual Health Res 2002;12:1284 99. 12448672 10.1177/1049732302238251

